# Who Were We? Exploring French Past Group Prototypes

**DOI:** 10.5964/ejop.7507

**Published:** 2023-08-31

**Authors:** Haifat Maoulida, Isabel Urdapilleta, Julie Collange, Jean Louis Tavani

**Affiliations:** 1 Laboratoire de Psychologie et d’Ergonomie Appliquées (UMR_T 7708), Université de Paris, Paris, France; 2 Laboratoire de Psychologie et d’Ergonomie Appliquées (UMR_T 7708), Université Gustave Eiffel, Paris, France; 3 Laboratoire Parisien de Psychologie Sociale (EA 4368), Université Paris 8 – Vincennes-Saint-Denis, Paris, France; 4 Laboratoire Cognitions humaine et artificielle (CHArt - EA 4004), Université Paris 8 - Vincennes-Saint-Denis, Paris, France; Heriot-Watt University, Edinburgh, United Kingdom

**Keywords:** prototype, collective memory, group dynamics, social identity, collective continuity

## Abstract

Groups have cognitive existence through the prototype of the group (Haslam et al., 1995; https://doi.org/10.1002/ejsp.2420250504). Past group prototypes then refer to the most representative characteristics that define the group in these previous states. We suppose, as collective events might have different versions associated with different valences (Zaromb et al., 2014; https://doi.org/10.3758/s13421-013-0369-7), this might also be the case for prototypes also held in the collective memory (Halbwachs, 1950; http://dx.doi.org/doi:10.1522/cla.ham.mem1). After highlighting different facets of the past (Study 1) or not (Study 2), we used the “free association method” (Lo Monaco et al., 2017; https://doi.org/10.1111/jtsb.12124; Vergès, [1992], L’évocation de l’argent. Bulletin de Psychologie, 45(4–7), 203–209). Yet, this research explored the content of past prototypes associated with different elements of French collective memory: the French during the Second World War (Study 1, N = 301), and French people in 18th century (Study 2, N = 354). Results suggest the existence for each of these periods of a “two-sided” prototype, i.e., a positive vs. negative-valence prototype. The implications of the existence of these “two-sided” prototypes, the implication of collective continuity perceived for each of them and avenues for future research will be discussed.

In many societies, we can observe that individuals and the groups to which they belong refer in their speech to the past to explain or justify the facts observed in their social world. Thus, we observe a form of universal naive thought according to which, individuals and groups, but also the relationships that they maintain, are shaped by their past. While this legitimacy of the link between past and present facts can be questioned, the use of past facts to justify the present, in the form of everyday and naive thought, never seems to be called into question. So, we construct ourselves as individuals from what we have been, and the same would be true for groups (Gkinopoulos, 2017; Licata & Klein, 2005). Therefore, we must consider that a group has a set of representations of the different objects of the past, relevant to the said group (e.g., events, characters), but also a set of representations of what it was previously (Condor, 2006; Liu et al., 2005). The representations of these past members are contained within the collective memory. The principal aim of this study is therefore to explore the content of this unexplored specific part of collective memory.

## Collective Memory

The French sociologist Maurice Halbwachs (1947, 1950, 2015), continuing the work of Durkheim (1912), was the first to conceptualize the term, 'Collective memory' (Misztal, 2003). Most definitions of it refer to the idea that collective memory is a set of representations and beliefs relating to the past (Jedlowski, 2001; Licata et al., 2007). This memory would be then shared and emerge from social interaction (Jedlowski, 2001). Collective memory can therefore be thought of as a social representation of a group’s past.

Through the stories shared by individuals, groups develop a common and shared representation of their past. Thus, through these memories, groups forge and define themselves and when they call on them, they redefine and update themselves in relation to present interests. It is therefore at the heart of the construction of its identity, of its standards and of its values, of the relationships maintained internally or externally (Liu & Hilton, 2005). This is one of the reasons why the group's past will play a crucial role by sometimes creating in the present a break with the unrewarding episodes of the past, and sometimes identifying it as in continuity with prestigious events from the past of this same group (Licata & Klein, 2005).

While research has focused on the work of reconstructing collective memory in the present, the influence of the past on the present seems to have aroused less interest. One of the explanations lies in the difficulty of studying a past which each time it is recalled undergoes changes. Nevertheless, the theoretical approach to social representations provides us with tools that allow us to study this past, which weighs so heavily on the present.

## Social Representations of the Past

Social representations are more generally considered as forms of knowledge about the world, emerging from social interactions (Jodelet, 2002; Rouquette, 2003). Among other things, they allow individuals to understand and give meaning to social objects and phenomena, including their own history (Lo Monaco et al., 2016). The body of knowledge about the past, when linked to a social group, is another way of defining collective memory (Tavani, 2012). These social representations of the past, or more broadly, these collective memories, are necessary to preserve a sense of group continuity and to cultivate values and norms that prescribe behaviors within a group and between a given group and external groups (Bobowik et al., 2018). Among the social representations that forge groups are those of its history.

These social representations of history are multiple (i.e., coexistence in a given society of different versions of memories of its history which come into interaction). They can just as well be hegemonic—i.e., consensual throughout society—as controversial—i.e., contradictory between different groups (Liu et al., 2005). Social representations of history include elements shared within a group (Moscovici, 1988) which allow people to think and act as a whole, through institutionalized normative imperatives, requiring a hegemonic social representation. Achieving such hegemony can be difficult, given the multiplicity of historical representations (possessed by the sub-groups that make up the largest social group) or even testimonies (environmental or verbal) of the past, of the mass media or of political interests (Liu et al., 2005). Indeed, collective memory seems to carry multiple and contradictory visions of the past which might interact and influence each other in the present (Liu et al., 2005). An example is the representations of colonialism carried which can be mapped through two main dimensions: the exploitation (of the resources of the country and of the indigenous peoples) and the development (of infrastructures and health systems (Figueiredo et al., 2018). The question then arises of knowing how these different representations are distributed in our society. Are they the marker of representations of two distinct social groups or can they coexist within the same whole?

## Content of the Collective Memory: The Notion of a Past Prototype

Collective memory therefore includes both the events of the past, the members of the groups that made it up, and the social representations of the history of a group containing descriptive elements (Moscovici, 1988). These past members are embodied both by the historical figures who have marked its history and by a form of prototype of the members who have made up the group over time. The prototype is defined as the representation of the average individual displaying all the representative characteristics of the members of a group at a given time (Hogg & Hardie, 1992). Even though some authors theorize prototypicality as a set of characteristics, fixed, shared by all (Hogg & Hardie, 1992; Hogg & Reid, 2006), this point of view is not unanimous. Thus, the extent to which a stimulus will be representative of a category—i.e., prototypical—is considered in the theory of self-categorization (Turner et al., 1987) as dependent on the characteristics of the social context in which the categorization is made (Haslam et al., 1995). Thus, Haslam et al. (1995) showed that a target is perceived as being more prototypical of a group when intergroup differences have been highlighted, i.e., when it is extreme in its group. This view allowed Haslam et al. (1995) to conclude that prototypicality is a property of the individual as a representative of a group in its context rather than as an isolated individual. But an alternative to consider would be that a prototype is associated with an event, a past culture or episode, and depends not only on the present context in which it is invoked, but also on the past context in which it emerged.

In short, the identity characteristics of a group would come together in the form of prototypes (“who we are”) and function as tools of action (“what we have to do”). So just as different situations call for different actions, they call for different stereotypes or prototypes (Klein & Licata, 2003; Rabinovich & Morton, 2012). This hypothesis that we formulate allows, among other things, two approaches, i.e., fixed and situational, which seem to be opposed, to be reconciled. It legitimizes the exit from a synchronic perspective which does not allow us theoretically to consider the past anchoring of prototypes and their study, in favor of a diachronic perspective, which considers, among other things, the possibility of a link and permanence over time. We should add that in order to understand prototypes and possibly study their impact, it is necessary to consider that, like other elements of collective memory, past prototypes are valence-carriers (Topcu & Hirst, 2020).

## Emotional Valence of Collective Memory

Collective memories form a network of feelings and thoughts (Halbwachs, 1950); they have an emotional resonance in today's societies with the development of technologies and the existence of a larger social network (Gong, 2001) which leads to increased sharing and intensification of group emotions. Indeed, memories in collective memory are not neutral and generally carry a positive or negative valence (Cyr & Hirst, 2019). This valence is linked to the experience of the members of the group during the event (Halbwachs, 1950). Events in collective memory can thus be classified according to their valence with, on the one hand the episodes related to a positive emotion, such as pride, love or joy, and on the other hand, the episodes related to a negative emotion, like shame, guilt or anger (Topcu & Hirst, 2020). Note that the same event can be represented both negatively and positively (Cyr & Hirst, 2019). This is the case with colonization: the positive representation highlights the civilizing benefits of colonization, while the negative representation emphasizes the dehumanizing aspect (Cabecinhas & Feijó, 2010; Figueiredo et al., 2018). Likewise, two nations can remember World War II differently: Americans remember the D-Day landings as the most important battle of the war, while for the Russians, the most important one is the Battle of Leningrad. (Brown et al., 2012; Wertsch, 2002). This dual viewpoint is also found in the role played (i.e., winner, loser, neutral) by a nation during this same period (Giner-Sorolla et al., 2021). Then, we assume that the past relationships of groups or past social status (i.e., place occupied by a group in the hierarchy of groups existing within their society (Dover et al., 2016; Sachdev & Bourhis, 1987), can make it possible to activate, from one group to another or within the same nation, several prototypes for the same event or period, prototypes which will be present to a greater or lesser extent from one member to another of the group. The perception according to which the ancestors of the group were advantaged/disadvantaged or had a low or high status in this past society (past social status) would be a determining variable, in addition to that of the valence of the memory, for identifying the type and the content related to the past prototype.

## Presentation of the Research

This research aims to determine from memories in the French^1^1Two preliminary studies were carried out to determine the memories most present in the French collective memory. The results are available in the Supplementary Materials. collective memory what are the characteristics of the members of the “French” group associated with each of them. More precisely, it will be a question of determining for each of the memories studied what the prototypes of the members of the in-group are. Using the free association method (Abric, 1994; Deschamps, 2003, 2005; Vergès, 1992) and from factorial analyses of multiple correspondences (Lo Monaco et al., 2017), we are working on studying these past prototypes. Indeed, within the framework of the study of social representations, associative tests aim to reveal traces of collective memory and to reason about the structure of the latter (Flament & Rouquette, 2003), as cited in (Dany et al., 2015).

As a reminder, although the theory of social identity has highlighted the existence of prototypes (e.g., Bartel & Wiesenfeld, 2013; Hogg et al., 2017; Hogg & Hardie, 1992), the study of these prototypes in the context of collective memory has not been studied. It seems important to us to identify the prototypes to which individuals refer when they remember, for example, the French during the World War II (Study 1), or even the French during the 18th century (Study 2). It is therefore a question here of studying the various past prototypes coexisting according to the valence of the memory. So, we assume that there is a positively and a negatively valenced prototype for each of these memories. Regarding memories associated with World War II (Study 1), a prototype of French-resistance (positive valence) would coexist with that of French-collaborator (negative valence). Concerning memories linked to the French 18th century (Study 2), a prototype of the French Revolutionary (positive valence), would coexist with a prototype of the French Colonizer/Slaver (negative valence). We hypothesize that respondents’ past social status has an impact on the salience of one or other of these prototypes. Thus, the descendants of members of the “colonized past” group such as participants of Caribbean origin would activate the French Colonizer/Slaver prototype while descendants of settlers (in mainland France) would activate the prototype of “French Revolutionary”.

We present two studies that aim to study these different memories and the prototypes associated with them.

## Study 1: Past Prototypes of the French During World War II

The objective of this first study was to explore the content of the past prototype associated with the World War II (WWII), by differentiating the two mnemonic facets of this event which are distinguished by the emotions they arouse (i.e., pride vs shame). To highlight the content of these prototypes, we wanted to question participants on the characteristics associated with French people who lived during this period.

### Method

#### Participants

Three hundred and sixty-four participants from the general population, all of French nationality (21.43% men and 78.57% women), voluntarily participated in this study (*M* = 26.03 years, *SD* = 10.58 years). The study took the form of a questionnaire, available online and distributed on social networks. The participants were assigned randomly to one of the three experimental conditions^2^2All the psychometric analyses and the material used are available in the Supplementary Materials.

#### Procedure

After filling in the socio-demographic information, the participants were invited to read a short (fictitious) text from the popular journal “History”. This short text, operationalizing the experimental conditions (cf. Appendices A to C in the Supplementary Materials), described the fictitious results of recent historical studies which highlight the different facets of the French during the World War II, namely resistance, collaboration, or a neutral. Then, using the free association method (Vergès, 1992), the participants indicated four words or expressions that spontaneously came to their mind when they thought of the French of that time. Then, they assessed the perceived continuity between their productions (i.e., characteristics associated with the French during WWII) and the French today. They then indicated the emotions felt towards the French of the same period. Finally, the participants completed a measure of identification with the French ingroup, a measure of temporal orientation, and a measure of perception of collective continuity.

#### Measures

For each study, after running general and item measures of sample adequacy and Bartlett’s sphericity test, a separate principal component analysis (with parallel analysis as a criterion; Horn, 1965) was performed. Each measure used a 7-point Likert-type scale ranging from 1 (Totally disagree) to 7 (Strongly agree).

##### Perceived Continuity With the French During World War II

To assess the perceived continuity between the prototype of the past group and the present prototype, the participants had, for each of the words they had produced, to say to what extent the 4 terms associated with the French of the past still characterized the French of today (α = .840).

##### Emotions Felt Towards the French of the Past

The participants indicated the intensity of their feelings towards France and its inhabitants during the World War II relating to 12 emotional states, taken from the work of (Smits et al., 2002). The first component brought together six positive emotional states: *pride, nostalgia, tenderness, joy, gratitude, melancholy* (α = .790). The second grouped together negative emotional states: *shame, regret, disgust, fear* and *ingratitude* (α = .740). The mixed item (nostalgia) was deleted because it did not saturate sufficiently on any of the components.

##### Identification With the French Ingroup

We measured, using five items, adapted from Doosje et al. (1998), the participants’ attachment to France and their identity as French. The identification score had a good internal consistency (α = .830).

##### Temporal Orientation of Individuals

We translated and used the scale of (Sharma, 2010) which makes it possible to assess individuals’ disposition to orient themselves more towards the past (dimension entitled “tradition” by the author, α = .69) or the present and the future (dimension entitled “prudence”, α = .82).

##### Perception of Collective Continuity

By adapting items from previous studies, cf. (Sani et al., 2007), we established a measure of dispositional temporal continuity. The latter refers to the general tendency of the individual to feel themselves to be in continuity with the history (narrativist continuity, α = .79), values and traditions of the group (essentialist continuity, α = .80).

### Results

#### Corpus Description

In total, 1784 terms for the three conditions combined were used to describe the French of the past (Collaboration: *N* = 584, Resistance: *N* = 605, Neutral: *N* = 589). We then proceeded to a descriptive analysis of certain aspects of the corpus, namely the number of responses produced, the number of response types, the number of hapaxes and the diversity and scarcity indices. Diversity is the ratio of different types of responses to the number of types of responses potentially produced (that is, the number of associative responses produced by the number of subjects per group). Rarity refers to the proportion of hapax in the corpus (element that has only been cited once). The index is calculated as follows: ratio between the number of hapax over the number of types, e.g., Flament & Rouquette, 2003; Tavani, 2012. These indices are presented in Table 1. The diversity and rarity indices were relatively similar from one condition to another, so we can conclude that the representational sharing between individuals is the same, for each type of prototype.

**Table 1:** Description of the Past Prototype Corpus Associated With WWII

Answers	General	Neutral	Resistance	Collaboration
Number	1784	589	605	585
Type	558	250	265	259
Hapax	336	170	180	173
Diversity	31.28%	42.44%	43.80%	44.27%
Rarity	60.22%	68.00%	67.92%	66.80%

We have identified, for each condition, the ten words most used by participants. In general, the words “Resistance” and “Collaborators” appear most often in each of their respective conditions. To better understand the differences between collaboration, resistance, and neutral conditions, we performed a Multiple Correspondence Factor Analysis on the corpus.

#### Factorial Analyses of Correspondences.

We kept the types of responses with a frequency equal to or greater than 10. This threshold is determined arbitrarily as indicating a high frequency of occurrence which refers to central representational elements (Flament & Rouquette, 2003). All conditions combined, 24 types of responses were retained. We then produced a factorial analysis of multiple correspondences from a contingency table representing in rows the types of responses and in columns the different modalities of our independent variables.

**Figure 1:**
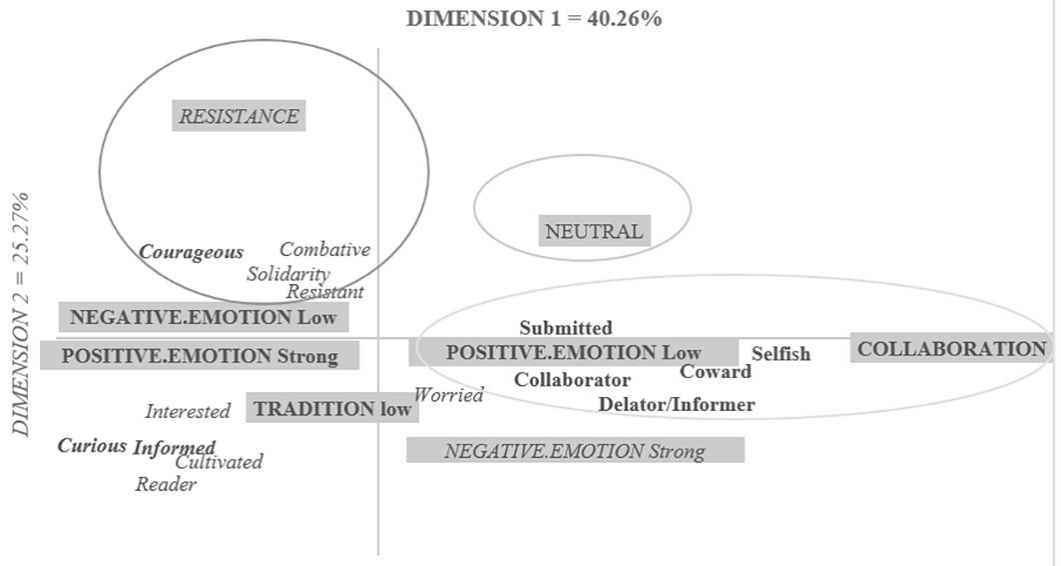
Representational Map of the Past Prototype WWII

The analysis highlights two factors that explain 67.51% of the inertia in the table (Factor 1 = 40.26%; Factor 2 = 27.25%, see Figure 1). Only the modalities of the variables and the types of responses contributing to the construction of the factors have been retained (Deschamps, 2003). The modalities of the variables contributing to the construction of Factor 1 are the conditions “collaboration” (CPF = 45.89%) and “neutral” (CPF = 12.99%), weak positive emotions (CPF = 6.73%), high positive emotional states (CPF = 9.21%), and weak negative emotional states (CPF = 8.37%). These modalities contribute 83.19% to the formation of Factor 1. Factor 2 is constructed by the conditions “Resistance” (CPF = 50.60%) and again “Neutral” (CPF = 23.40), the modality “tradition” of the variable temporal orientation at low level (CPF = 6.84%) and by strong negative emotions (CPF = 5.50%). In total, these modalities refer to a total contribution of 95.3%. The terms contributing to Factor 1 are in bold on the graph, those contributing to Factor 2 are in italics. The modalities of IVs are boxed in gray.

The first factor opposes the collaboration condition to neutral. The terms most closely related to the collaboration variable are *cowardly, selfish, informer/denouncer*, while the term neutral is not strongly attached to any term. The second factor opposes the resistance to neutral condition. As before, the neutral condition is isolated from all the terms, while the resistance condition is associated with the terms: *combative, resistant, supportive*.

The results of this factor analysis confirm those obtained from the frequency analysis, while bringing to light a set of differentiated characteristics associated with past prototypes that are positive and negative. Note that we do not observe characteristics associated with a prototype of the past that is not associated with a given valence, in other words we do not observe a neutral prototype.

## Study 2: Past Prototypes of 18th Century French People, Influence of Past Social Status

The aim of this study is to explore the content of the past prototype associated with the French 18th century by differentiating two subgroups with different past social status: the French-Caribbean (descendants of slaves/colonized) and the French-Metropolitan (descendants of slavers/settlers). We hypothesize that this century represents a double memory from an emotional point of view (of the emotions aroused by the recall of the past collective memory) which would be manifested by the presence in the French collective memory of two separate prototypes. Thus, the French-Caribbean (past minority status) would perceive this period of history mainly through the prism of colonization and slavery, relating this period to negative emotions. For the French-Metropolitan (past majority status), this period would be linked to rather positive emotions, linking this century to the revolutionary period and to the Enlightenment.

### Method

#### Participants

Three hundred and fifty-four participants from the general population (with 26.84% men) voluntarily completed this study; 236 participants identified themselves as French-Metropolitan (*M =* 26.41 years, *SD =* 10.13 years) and 118 participants identified themselves as French-Caribbean (*M =* 31.89 years, *SD =* 11.95 years). The study took the form of an online questionnaire, distributed on social networks, mainly targeting groups identifying themselves as Caribbean.

#### Procedure

The participants, after filling in the socio-demographic information, were invited to state the terms they associated with 18th century France (cf. Appendix D in the Supplementary Materials). Then, we presented them with a short version of the perception of collective continuity scale and a measure of the emotions felt towards the French during this past period. Then, they had to indicate whether they saw themselves more as French-Metropolitan, French-Caribbean or other. Finally, they completed a measure of identification with the subgroup they had indicated.

#### Measures

##### Perception of Collective Continuity

The scale was the same as that used in Study 1. The first component referred to essentialist continuity (α = .818) and the second to narrative continuity (α = .797).

##### Emotions Felt Towards the French of the Past

The participants indicated the intensity of their feelings towards France and the inhabitants of the 18th century by measuring the emotional states used previously (positive α = .807 and negative emotional states, α = .853).

##### Identification With the Ingroup

This was measured based on the same items used previously, items that we adapted for the French-Caribbean group to measure their attachment to their Caribbean identity (α = .897).

### Results

#### Corpus Description

A total of 1,424 terms for both conditions were produced to describe the 18th century French (French-Metropolitan: *N* = 951, French-Caribbean: *N* = 473). The description of the corpus is presented in Table 2. The diversity and rarity indices are quite different from one condition to another, which gives us the first indications of a representational divergence between individuals of different social status.

**Table 2:** Description of the Past Prototype Corpus Associated With the 18th Century

Answers	French	Metropolitan	Caribbean
Number	1424	951	473
Type	388	293	183
Hapax	263	207	127
Diversity	27.25%	30.81%	38.69%
Rarity	18.47%	21.77%	26.84%

To analyze the differences between the two past social statuses, we carried out a factorial correspondence analysis on the corpus.

#### Factorial Analyses of Correspondences

This was carried out on a contingency table representing in rows the types of responses and in columns the different modalities of our independent variables: social status (French-Metropolitan, French-Caribbean), sex (man, woman), perception of collective continuity. (essentialist narrative), and emotion (positive, negative) towards the French of the past, identification with the ingroup (high, low) for each of the corpus.

**Figure 2:**
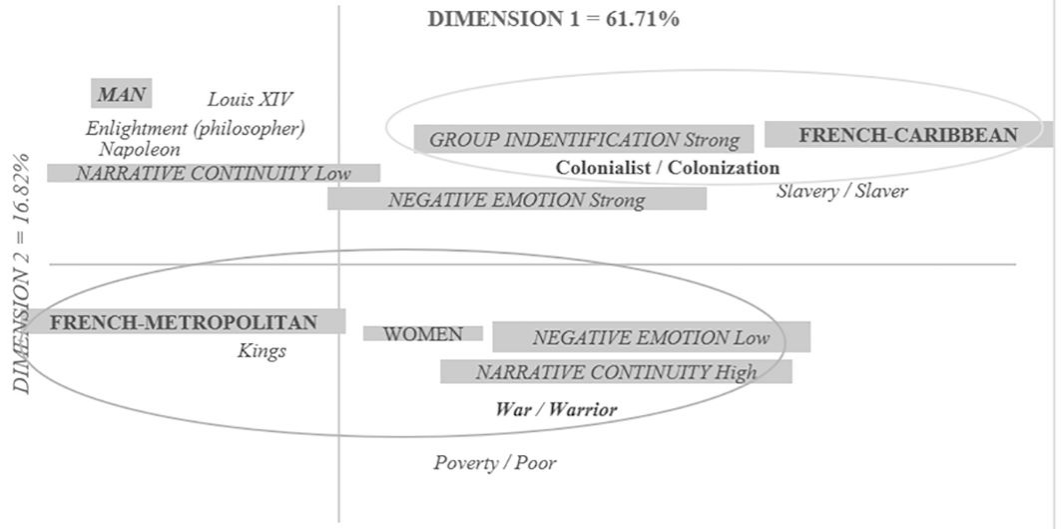
Representational Map of the French Prototype of the 18th Century

The analysis highlights two factors that explain 61.71% of the inertia in the table (Factor 1 = 61.71%; Factor 2 = 16.82%, see Figure 2). The modalities of the variables contributing to the construction of Factor 1 are the status “French-Caribbean” (CPF = 38.72%) and “French-Metropolitan” (CPF = 16.96%). These modalities contribute 55.68% to the formation of Factor 1. Factor 2 is constructed by the “Male” sex (CPF = 9.29%); narrative continuity at a low (CPF = 8.74%) and high (CPF = 13.65%) level, weak (CPF = 9.17%) and strong (CPF = 7.76%) negative emotions and strong in-group identification (CPF = 5.80%). In total, these modalities refer to a total contribution of 54.41%.

The first factor mainly opposes past social statuses by comparing the French-Metropolitan prototype to that of the French-Caribbean. The terms most closely linked to the French-Metropolitan variable are those of *war/warrior*, while for the French-Caribbean they are the terms *colonization/colonialist.* The second factor contrasts, on the one hand, a strong negative emotion coupled with a weak narrative continuity (see Figure 2, and on the other, a weak negative emotion associated with a strong narrative continuity, a strong identification with the in-group and the male sex. The first association is linked to the terms *poverty/poor, war/warrior, kings*, while the second association of variables is linked to *the Enlightenment (philosopher), Napoleon, Louis XIV*.

## General Discussion

### Appraisal

In the present research, we proposed reconciling the fixed (Hogg & Reid, 2006) and situational (Haslam et al., 1995) approaches of the study of the prototype by considering that a prototype would be fixed during its construction, but that depending on the context, the members of a given group could use one prototype rather than another. Thus, the group would establish a set of characteristics determining this prototype at a given time, without this excluding the possibility of creating several. So, we hypothesized that there would be one positive valence and one negative valence prototype for each of these memories. The objective of this study was to respond to this hypothesis based on the study of two French collective memories: World War II (Study 1) and France of the 18th century (Study 2).

Indeed, World War II was a “two-sided” memory, one positive (Resistance), one negative (Collaborator), and each of these facets has unique, specific characteristics associated with it.

In Study 2, analyses confirmed that French-Caribbeans (past minority status) mostly adhere to a rather negative French past prototype such as “slaver” or “colonizer”, whereas French-Metropolitans (past majority status) adhere to a positive French past prototype such as “fighter” or “believer”.

The results of these two studies confirm the idea that in the collective memory the prototypes would be associated with an emotional valence (positive *vs.* negative) and that these related past prototypes would be linked to past social status. However, in contrast to what previous studies have suggested (e.g., Sani et al., 2007; Smeekes & Verkuyten, 2015), in our studies ingroup identification did not impact prototype content. One explanation can be borrowed from the literature on collective guilt. Indeed, feelings of collective guilt for past ingroups’ transgressions depend on the type of ingroup identification, namely glorification or attachment (Bonnot et al., 2016). When national identification comes in the form of glorification, people are motivated to perceive their group as better and more worthy than other groups in order to maintain a positive image of their ingroup. Consequently, they would be more incline to minimize, or even deny the responsibility of their group, in their past harmful actions (Bonnot et al., 2016). In the attachment form, people feel connected and loyal to the group but may look critically at the group, and by extension at their involvement in past events (Bonnot et al., 2016). They would thus be more inclined to acknowledge the responsibility of the ingroup’s past transgression. Thus, future studies should examine how the type of identification might influence the construction of the ingroup prototype.

Overall, these results encourage a more systematic study of past group members’ representations. Future work should focus more on the exploration of prototypes, such as historical events and figures which have received the main attention in studies of collective memory (Coman et al., 2009).

### Limitations and Future Research

The arbitrary choice of the frequency threshold to be reached to be representative of the past group, as well as the limit given for the number of terms are all limitations to the choice of a measure based on the free association method. However, these limitations are not, in our opinion, enough to reject this method, but enough for us to consider how to improve it. We could take into account the hierarchy or rank of the elements reported by the participants (Dany et al., 2015; Lo Monaco et al., 2017).

The results of the studies presented here lead us to think that it would be necessary to devise experimental studies that would make it possible to further highlight these initial results. For example, using the characteristics of the various prototypes identified to build portraits of these French of the past. Then, we might study the effect of the perception of continuity with any of these past two-sided prototypes on different variables of interest in social identity. Indeed, in the introduction we mentioned the defining role that collective memory plays in group and social identity (Licata & Klein, 2005). The activation of these prototypes and the perception of continuity with them should have consequences for present attitudes, opinions and behaviors, related to intragroup (e.g., social judgment, perceived similarity, etc.) or intergroup relations (e.g., collective actions, rejection of outgroup, etc.). Some results of our current work point in this direction.

Moreover, in Study 1, we chose to highlight different aspects of WWII in France—i.e., resistance, collaboration or neutral—to examine how the prototype content of the French in would differ according those aspects of World War II. This experimental manipulation allowed us to ensure that each aspect or side of WWII would be salient for an equivalent number of participants—and consequently an equivalent number of verbal productions in each condition (positive *vs.* negative prototype). However, this procedure does not allow us to examine which aspects of WWII is the most salient in our sample, and therefore which prototype is more accessible. Thus, although the neutral condition could be considered as a control condition, future studies should investigate which prototype is more present within the French population and which factors could influence the presence of one prototype rather than another (e.g., family history, region in which they live, identification with the French group, etc.). Indeed, one could think that, in order to maintain a positive ingroup image, individuals would spontaneously produce a positive past prototype of the French (i.e., as resistant, especially those who are highly identified to France; Sahdra & Ross, 2007).

Furthermore, the predominance of one prototype over another among our participants could be one of the likely explanations for the lack of effect of collective continuity. We could hypothesize that the imposed recall prototype is not the predominant prototype of the participants interviewed. Thus, from one participant to another, the perception of continuity between this past and current prototype would vary and this effect of continuity on the content of the prototype could not be observed.

Finally, these works highlight the existence of a new form of collective continuity, the perception of continuity with past prototypes, i.e., the perception of a link between the members of a past, present, or future group; efforts to explore it should be continued. Indeed, this form of continuity might go beyond or even transcend any belonging to narrative or essentialist continuity theorized by Sani and collaborators (2007) and could perhaps be considered as a third form of collective continuity.

### Conclusion

The aim of this research was to introduce the notion of past prototype. We have explored the characteristics associated with the French of the past, (1) of the World War II and (2) of the 18th century and assumed a “two facets” prototype: one with a positive and one with a negative valence. So, the French of WWII could be “the French-Collaborator” or the “French-Resistant”); depending on past social status, the French of the 18th century could be “the Revolutionary” or “the Slaver/Colonizer”. Past prototypes and their content have been the subject of too few studies in the field of collective memory. The present research proposes a methodology, the free associations method, as an effective way to examine these prototypes. Moreover, the results of this research could have strong implications for intra-group and even inter-group relations. Indeed, collective memories define group identities and are a relevant criterion for social categorization (Tavani et al., 2017). Thus, if past prototypes constitute norm of remembering, deviance to the most dominant prototype (positive *vs.* negative valence) might constitute a criterion for excluding someone from the ingroup. Additionally, as shown by Maoulida et al. (2021), perceived continuity to a positive versus a negative past influence attitudes towards outgroups. Future studies should thus further examine the influence of perceived collective continuity regarding past prototypes, i.e., continuity between the group past and present identity, on intragroup variables, e.g., collective self-esteem, social categorization, and intergroup ones.

## Supplementary Materials

The supplementary materials provided are data, material, appendices, and analyses that support the findings of this study (for access see Index of Supplementary Materials below).



MaoulidaH.
UrdapilletaI.
TavaniJ. L.
 (2020). Who we were? Exploring French past group prototypes"
[Data, materials, analyses]. PsychOpen. https://osf.io/mbfep/
10.5964/ejop.7507PMC1050819937731752

MaoulidaH.
UrdapilletaI.
CollangeJ.
TavaniJ. L.
 (2023). Supplementary materials to "Who were we? Exploring French past group prototypes"
[Appendices]. PsychOpen. 10.23668/psycharchives.13166
PMC1050819937731752

## Data Availability

The data and material, including appendices, used for this study as well as additional analyses are freely available and can be found in the Supplementary Materials.
